# Clonal Dissemination of Antifungal-Resistant *Candida haemulonii*, China

**DOI:** 10.3201/eid2903.221082

**Published:** 2023-03

**Authors:** Xinfei Chen, Xinmiao Jia, Jian Bing, Han Zhang, Nan Hong, Yun Liu, Haiyang Xi, Weiping Wang, Zhiyong Liu, Qiangqiang Zhang, Li Li, Mei Kang, Yuling Xiao, Bin Yang, Yulan Lin, Hui Xu, Xin Fan, Jingjing Huang, Jie Gong, Juan Xu, Xiuli Xie, Wenhang Yang, Ge Zhang, Jingjia Zhang, Wei Kang, He Wang, Xin Hou, Meng Xiao, Yingchun Xu

**Affiliations:** Peking Union Medical College Hospital, Chinese Academy of Medical Sciences and Peking Union Medical College, Beijing, China (X. Chen, X. Jia, H. Zhang, J. Huang, X. Xie, W. Yang, G. Zhang, J. Zhang, W. Kang, M. Xiao, Y. Xu);; State Key Laboratory of Complex Severe and Rare Diseases and Beijing Key Laboratory for Mechanisms Research and Precision Diagnosis of Invasive Fungal Diseases, Beijing (X. Chen, X. Jia, H. Zhang, J. Huang, X. Xie, W. Yang, G. Zhang, J. Zhang, W. Kang, M. Xiao, Y. Xu);; Fudan University, Shanghai, China (J. Bing, Q. Zhang, L. Li);; Nanjing University School of Medicine, Nanjing, China (N. Hong, H. Xi, W. Wang);; Changhai Hospital, Shanghai (Y. Liu);; Southwest Hospital Affiliated the Third Military Medical University, Chongqing, China (Z. Liu);; Sichuan University, Chengdu, China (M. Kang, Y. Xiao);; The First Affiliated Hospital of Fujian Medical University, Fuzhou, China (B. Yang, Y. Lin);; The First Affiliated Hospital of Zhengzhou University, Zhengzhou, China (H. Xu);; Capital Medical University, Beijing (X. Fan);; Chinese Center for Disease Control and Prevention, Beijing (J. Gong, J. Xu);; Dynamiker Sub-Center of Beijing Key Laboratory for Mechanisms Research and Precision Diagnosis of Invasive Fungal Disease, Tianjin, China (H. Wang);; Peking University Third Hospital, Beijing (X. Hou)

**Keywords:** *Candida haemulonii*, clonal dissemination, antifungal resistance, China, whole-genome sequencing, multidrug resistance, antimicrobial resistance, fungi

## Abstract

*Candida haemulonii*, a relative of *C. auris*, frequently shows antifungal resistance and is transmissible. However, molecular tools for genotyping and investigating outbreaks are not yet established. We performed genome-based population analysis on 94 *C. haemulonii* strains, including 58 isolates from China and 36 other published strains. Phylogenetic analysis revealed that *C. haemulonii* can be divided into 4 clades. Clade 1 comprised strains from China and other global strains; clades 2–4 contained only isolates from China, were more recently evolved, and showed higher antifungal resistance. Four regional epidemic clusters (A, B, C, and D) were identified in China, each comprising ≥5 cases (largest intracluster pairwise single-nucleotide polymorphism differences <50 bp). Cluster A was identified in 2 hospitals located in the same city, suggesting potential intracity transmissions. Cluster D was resistant to 3 classes of antifungals. The emergence of more resistant phylogenetic clades and regional dissemination of antifungal-resistant *C. haemulonii* warrants further monitoring.

The first case of human infection caused by the yeast *Candida haemulonii* was reported in 1984 (*1*). Recent research has indicated that the previously recognized *C. haemulonii* species is actually a species complex comprising 4 phylogenetically closely related species, *C. haemulonii*, *C. duobushaemulonii*, *C. pseudohaemulonii*, and *C. vulturna* (*1*,*2*). The emerging, highly problematic pathogen *C. auris*, which is also a closely related species of the *C. haemulonii* complex, was first reported in Japan in 2009; it has attracted widespread attention worldwide owing to its multidrug resistance and capacity to cause nosocomial outbreaks (*3*–*5*). Because the overall prevalence of *C. haemulonii* sensu stricto remains low worldwide, less attention has been paid to this species. Like *C. auris*, *C. haemulonii* exhibits notable resistance to various classes of antifungal agents, including azoles and amphotericin B (*6*–*8*), and some reports have described nosocomial outbreaks caused by *C. haemulonii* (*9*). However, although *C. haemulonii* s.s. has been discovered in a broad range of wild environmental and animal sources (*10**–**15*), it has not been isolated from a hospital environment.

Molecular methods play important roles in clinical mycology, including laboratory diagnostics, taxonomic investigations, phylogenetic analysis, and confirmation of outbreaks (*16*). Previous studies on the *C. haemulonii* complex have applied methods such as sequencing of the rDNA internal transcribed spacer (ITS) region, amplified fragment-length polymorphism, and random amplified polymorphic DNA; however, the discriminatory powers of those methods are limited and only capable of assigning isolates to the species level (*2*). Whole-genome sequencing (WGS) provides a high-resolution alternative. In fact, WGS-based genomic analysis has assisted in tracing the phylogenetic evolution and dissemination of *C. auris* globally (*17*), confirming nosocomial transmission of *C. auris* in healthcare facilities (*5*,*18*,*19*), and analyzing potential antifungal resistance mechanisms (*20*–*22*).

The global phylogeny of *C. haemulonii* remains uncharacterized. The China Hospital Invasive Fungal Surveillance Net (CHIF-NET) program identified several regional clustered cases (n >5) in China caused by *C. haemulonii*; however, the overall prevalence of this species remained low (0.8%) (*23*). We performed WGS-based analysis of 94 *C. haemulonii* strains, 58 isolates collected from 23 hospitals by the CHIF-NET study over 8 years in China and 36 previously published international strain genomes (*24*). The primary goal of our study was to illustrate the phylogenetic character of this species worldwide and determine the population relatedness of regional cluster cases in China. In addition, we sought to predict major antifungal resistance mechanisms using bioinformatic analysis. Our study was approved by the Human Research Ethics Committee of the Peking Union Medical College Hospital (protocol S-263).

## Materials and Methods

**Figure 1:**
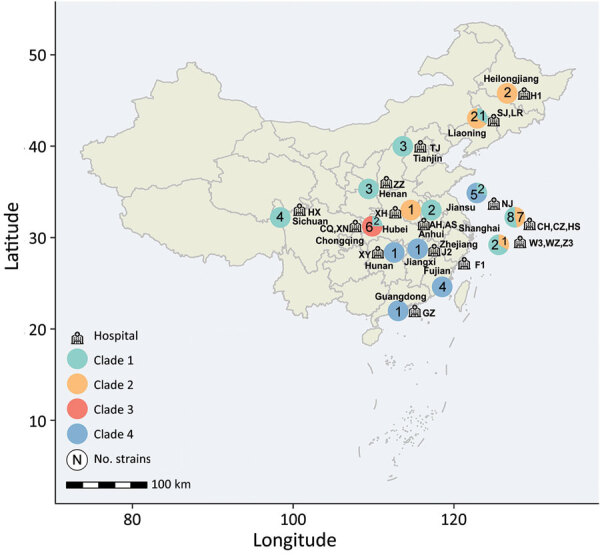
Regional distribution of 58 invasive infections caused by *C. haemulonii* in China during 2010–2017, collected from the China Hospital Invasive Fungal Surveillance Net study. Province names are listed, and hospital locations are marked by icons; the abbreviation codes of hospitals are listed next to each location. The pie charts adjacent to the province names indicate the number of isolates collected; phylogenetic clades are labeled in different colors.

We examined 58 nonduplicated clinical *C. haemulonii* isolates collected from 23 hospitals distributed across 15 provinces in China during August 2009–July 2017 (Figure 1). Of those strains, 31 had been previously reported (*7*). We also included publicly available genomic data for 36 international *C. haemulonii* strains, obtained from the National Center for Biotechnology Information Sequence Read Archive.

Of the strains from China, 69% (40/58) were isolated from the blood and 13.8% (8/58) from the cerebrospinal fluid. The remaining strains were isolated from venous catheters (8.6%, 5/58), secretions (3.4%, 2/58), tissue fluid (1.7%, 1/58), ascitic fluid (1.7%, 1/58), and drainage (1.7%, 1/58) (Appendix 1 Table 1). Samples came from from patients in medical wards (53.4%, 31/58), surgical wards (22.4%, 13/58), intensive care units (22.4%, 13/58), and emergency departments (1.7%, 1/58).

The international strains were isolated from 3 continents: 18 from South America (Venezuela, n = 7; Colombia, n = 11), 17 from North America (United States, n = 13; Panama, n = 4), and 1 from Asia (Israel, n = 1). Of the strains, 94.4% (34/36) were from humans (blood, wounds, bone bronchial wash, foot, vaginal secretion, catheter, urine, or peritoneal fluid), 2.8% (1/36) from animals (fish), and 2.8% (1/36) with no source information (Appendix 1 Table 1).

We identified all strains by using matrix-assisted laser desorption/ionization time-of-flight mass spectrometry and ITS sequencing (Appendix 2). We evaluated in vitro susceptibility and performed WGS to explore the molecular features of the isolates. Raw genome reads are available from the National Center for Biotechnology Information (BioProject no. PRJNA827237). 

## Results

### Collection of Isolates

We identified all strains as *C. haemulonii* by using Autof MS 1000 (Autobio Diagnostics Co., Ltd; https://en.autobio.com.cn) and Vitek MS (bioMerieux; https://www.biomerieux-usa.com/). The phylogenetic tree based on rDNA ITS region sequences revealed that CHIF-NET strains clustered with *C. haemulonii* CBS5149^T^ rather than other species within the *C. haemulonii* species complex. 

### *C. haemulonii* Genome Highly Conserved

We performed single-nucleotide polymorphism (SNP) calling for all 94 isolates. Although derived from vast international geographic regions, we found *C. haemulonii* genomes to be highly conserved. We found 6,807 SNPs among the 94 *C. haemulonii* genomes, which was a considerably smaller number than that first reported for *C. auris* (119,188 SNPs) (*4*). The pairwise SNP differences among all international strains ranged from 6 to 553 (median 269). SNP differences between Chinese and international isolates ranged from 4 to 653 (median 333), and pairwise SNP differences between different Chinese strains ranged from 6 to 581 (median 297).

### Four Phylogenetic Clades Identified Worldwide

**Figure 2:**
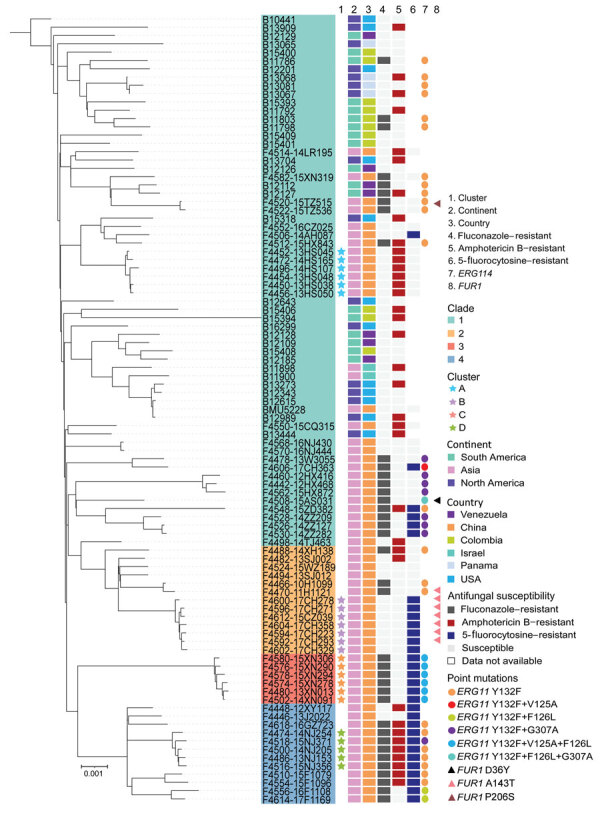
Maximum-likelihood phylogenetic tree constructed based on whole-genome single-nucleotide polymorphisms and phylogenetic clades in a study of antifungal-resistant *Candida haemulonii* in China. Information is labeled for each strain: geographic origin, antifungal susceptibilities for representative drugs of different classes (fluconazole, amphotericin B, and 5-fluorocytosine), and key amino acid substitutions related to antifungal resistance that were observed in genes encoding lanosterol 14-α-demethylase (*ERG11*) and uracil phosphoribosyltransferase (*FUR1*). The tree was rooted to strain B10441 (CBS5149), which is the most ancient *C. haemulonii* strain, identified in 1962 (from *Haemulon sciurus*). All remaining strains were isolated after 2010.

Fast hierarchical Bayesian analysis of population structure revealed that all strains could be divided into 4 major clades, and principal components analysis results clearly supported the presence of these 4 groups (Figure 2; Appendix 2 Figure 1). We classified 63 isolates (67%) as clade 1, 13 (13.8%) as clade 2, 6 (6.4%) as clade 3, and 12 (12.8%) as clade 4 (Appendix 1 Table 1). From the phylogenetic tree, we observed that clade 1 strains were widely distributed across vast geographic regions (Figure 1). In comparison, all isolates in clades 2, 3, and 4 were exclusively from China (clade 2, n = 13; clade 3, n = 6; clade 4, n = 12), and those 3 branches are suggested to have evolved from clade 1 in the phylogenetic tree. Of note, analysis of the mating-type locus showed that all 94 isolates were *MATα*. 

### Regional Clustered Cases Associated with Spread of Specific Clones

We observed several clustered regional cases. To investigate potential clonal spreads or outbreaks, we first concentrated on any hospital with ≥5 cases of *C. haemulonii* infections that occurred during the surveillance period. We found that the maximum pairwise SNP differences for isolates within the same clade from the same hospital were all <50 (33 SNPs for clade 1 in hospital HS, 28 for clade 2 in hospital CH, 34 for clade 3 in hospital XN, and 45 for clade 4 in hospital NJ). Except for isolates of clade 3 that were identified in only 1 hospital, the above differences were considerably less than the average intra-clade pairwise SNP differences of all isolates within the same clade, which were 301 SNPs for clade 1, 131 for clade 2, and 160 for clade 4. We therefore used a criterion of ≤50 SNPs for defining clonal clusters in our primary analysis. On the basis of those criteria, we identified 4 obvious clusters.

**Figure 3:**
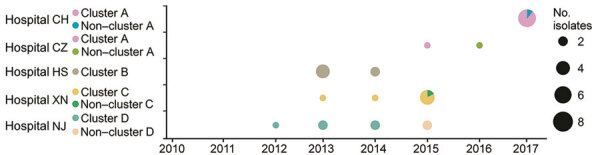
Distribution of 4 regionally disseminated *Candida haemulonii* clusters (clusters A, B, C, and D) in 5 hospitals in China. Isolates not belonging to the 4 major clusters were summarized as noncluster strains. Pie charts indicate number of isolates (indicated by size of pie) and distribution of clusters (distinguished by color).

We discovered cluster A, initially, in hospital CH in East China; 6 cases accounted for 85.7% (6/7) of the *C. haemulonii* infection cases found in that institution. Cluster A isolates belonged to clade 2, and SNP differences between any 2 cluster A strains ranged from 10 to 28 bp (median 21). Three strains were isolated from the surgical ward, 2 strains from the medical department, and 1 strain from the intensive care unit. Five strains were isolated from blood and 1 strain from cerebrospinal fluid. The remaining non–cluster A isolates from that hospital belonged to clade 1, which differed from the cluster A strains, ranging from 349 to 399 bp (median 398). Of note, 1 strain isolated from another hospital (hospital CZ, also located in hospital CH’s city) fell into cluster A (paired SNPs 13 to 22 versus CH cluster A strains), suggesting intra-city transmission of *C. haemulonii* from August 2016 through April 2017 (Figure 3).

Cluster B, belonging to clade 1, was detected in hospital HS in East China, comprising 6 cases, and the inter-cluster pairwise SNP differences ranged from 6 to 33 bp (median 20). Although hospitals HS, CH, and CZ were in the same city, cluster B diverged from cluster A (>258 bp differences). Four cluster B isolates were collected from the infectious department ward, 1 isolate was collected from the emergency department, and 1 isolate was collected from the intensive care unit. All strains were isolated from cerebrospinal fluid during October 2013–August 2014 (Figure 3).

Cluster C was detected in hospital XN in Southwest China and belonged to clade 3. Of the 7 strains isolated from hospital XN, 85.7% (6/7) were attributed to cluster C. The remaining isolate was from clade 3 but differed from other cluster C strains by 451–463 bp. Within cluster C strains, pairwise SNP differences ranged from 11 to 34 bp (median 28). All cluster C strains from hospital XN were isolated from the hepatobiliary ward; 3 of those strains were cultured from blood and catheter samples. The timeline for the isolation of cluster C isolates in hospital XN was >2 years (September 2013–October 2015) (Figure 3).

Cluster D isolates belonging to clade 4 were detected from hospital NJ in East China and comprised 5 cases. The number of SNPs in cluster D ranged from 19 to 45 bp (median 37). All 6 cluster D strains were isolated from the general surgery wards. As with cluster C strains, cluster D strains persisted in hospital NJ for >2 years (September 2013–November 2015) (Figure 3). Of interest, 2 additional non–cluster D strains from that hospital belonged to clade 1. Both strains were isolated from the nephrology ward (August 2016–November 2016), and pairwise SNP differences between the 2 strains were only 11, suggesting another potential nosocomial transmission.

In summary, 4 clonal dissemination events of *C. haemulonii* were identified in China. Moreover, evidence suggests the occurrence of an intra-city clonal spread caused by a multidrug-resistant clone.

### Notable Antifungal Resistance in *C. haemulonii*

Among the 94 isolates studied, only an international strain from Venezuela was reported to be resistant to caspofungin (MIC = 16 μg/mL) (*24*). All other strains remained susceptible to echinocandins. Of the isolates, 40.4% (38/94) were resistant to fluconazole and 21.2% (20/94) were resistant to voriconazole, including 24.5% (23/94) that were cross-resistant to the 2 azoles. The resistance rate in China was 56.9% (33/58) for fluconazole and 34.5% (20/58) for voriconazole, both rates higher than the rates among international strains, which were 13.9% (5/36) for fluconazole and 0% (0/36) for voriconazole. In comparison, only 9.6% (9/94) of the isolates were resistant to itraconazole, and only 1 isolate had a minimum inhibitory concentration ≥1 μg/mL for posaconazole. Nearly half (44.7%, 42/94) of the isolates were resistant to amphotericin B. Although data for 5-fluorocytosine resistance were not available for the 36 international strains, more than half (53.4%) of the 58 strains from China were 5-fluorocytosine resistant. Moreover, in China, 25.8% (15/58) of the isolates were multidrug resistant, including 15.5% (9/58) that were resistant to 3 classes of antifungal agents.

Antifungal resistance was associated with the clonal background of the strains. For instance, fluconazole resistance rates were above 80% for clade 3 (100%) and clade 4 (83.3%) strains, whereas only 30.2% of clade 1 and 46.2% of clade 2 isolates were fluconazole resistant (Appendix 1 Table 2). In addition, China clade 1 isolates exhibited a higher fluconazole resistance rate (51.9%) than the international strains (13.9%). The amphotericin B resistance rate of strains in clade 1 (49.2%) and in clade 4 (58.3%) were higher compared with other clades (<20%). The 5-fluorocytosine resistance rate was 100% in clades 3 and 4. Strains resistant to 3 classes of antifungals were mainly distributed in clade 4 (66.7%), including all cluster D isolates (Appendix 1 Table 2).

### Potential Resistance Mechanisms of *C. haemulonii*

We used the genome of strain BMU5228 as a wild-type sequence to annotate gene mutations in 25 known important antifungal resistance genes (Appendix 1 Table 3). Among the 33 fluconazole-resistant strains in China, 100% harbored the Y132F substitution in Erg11p (Table). We also found the Y132F substitution in 11.1% (4/36) of the international strains, and 2 of them were fluconazole resistant. We found 54.5% (18/33) of fluconazole-resistant strains in China harbored >1 of the substitutions V125A, F126L, and G307A (Table; Appendix 1 Table 3). We screened other genes reported to cause azole resistance and found that 6 cluster C strains had the substitution M589L in Tac1Bp, the transcriptional regulator of the efflux pump Cdr1. Our analysis of the distribution of copy number variations revealed that 13 (22.4%) strains in China had >1 copy of the *ERG11* gene, and those strains were all resistant to fluconazole (Appendix 2 Figure 2). For strains with >1 copy of *ERG11*, 6 strains were from clade 3 and 7 strains were from other clades (Appendix 2 Figure 2). Isolates with >1 copy of *ERG11* had significantly higher MICs against fluconazole than did isolates with 1 copy (p<0.05 by Mann–Whitney test). For 5-fluorocytosine, of the 31 resistant isolates, 25.8% (8/31) had noteworthy mutations in the *FUR1* gene, including 7 strains carrying the substitution A143T (all of which were cluster C) and 1 strain carried the substitution P206S. Although 42.6% (40/94) of the strains were resistant to amphotericin B, we observed no mutations of note in the previously described resistance-related genes, including *ERG2*, *ERG3*, or *ERG6* (Appendix 1 Table 3).

**Table:** Distribution of noteworthy Erg11p substitutions among 4 clades of *Candida haemulonii* strains studied as part of an investigation of antifungal-resistant *C. haemulonii*, China

Clade/ geographic origin	Erg11p substitutions				No. isolates
	Y132F	V125A	F126L	G307A	
Clade 1					
International	Y	N	N	N	8
China	Y	N	N	N	5
	Y	N	N	Y	7
	Y	Y	N	N	1
	Y	N	Y	Y	1
Clade 2					
China	Y	N	N	N	3
Clade 3					
China	Y	Y	Y	N	6
Clade 4					
China	Y	N	N	N	7
	Y	N	Y	N	2
	Y	N	N	Y	1

## Discussion

In recent years, the number of human infections caused by emerging pathogens has increased gradually (*24*). Among those pathogens, *C. haemulonii* and its closely related species *C. auris*, belonging to the family Metschnikowiaceae, have received great public attention because of their notable trends of antifungal resistance and capacity to cause nosocomial transmission (*5*,*9*,*25*).

Genome-based phylogenetic studies of *C. auris* have revealed that 5 distinct clades (I, II, III, IV, and V) are distributed in East Asia, South Asia, South Africa, and South America (*17*,*26*), whereas the population structure of *C. haemulonii* has not been previously defined. In this study, we found that *C. haemulonii* can also be divided into 4 phylogenetic clades. Rooted by the most ancient *C. haemulonii* strain B10441 (CBS5149) that was isolated in 1962, strains from clades 2–4 emerged more recently, with isolates identified exclusively in China and antifungal resistance observed more notably compared with clade 1. We found that 46.2% of clade 2 isolates were fluconazole resistant versus 30.2% in clade 1, all clade 3 isolates were cross-resistant to fluconazole and 5-fluorocytosine, and 66.7% of clade 4 isolates were resistant to 3 classes of antifungals.

Although *C. haemulonii* can be divided into 4 clades, the total number of SNPs identified in the 13.31 Mb whole genome of *C. haemulonii* was only 6,807 (<0.005%), which was considerably less than that in *C. auris*, which has a similar genome size (>210,000 SNPs in a 12.4 Mb genome) (*27*–*29*). When we compared the most ancient *C. haemulonii* strains identified to date (strain ID no. CBS5149, isolated from *Haemulon sciurus* fish in 1962) with the other strains in our study, the maximum genome sequence difference was only 384 bp. Those factors indicate that the genome of *C. haemulonii* is highly conserved. In some *Candida* species, mating can lead to an increase in genetic diversity, and opposite mating types have been observed in *C. auris* (*17*,*29*). However, all *C. haemulonii* isolates identified were of the same mating type (type α), and a sexual cycle has not been observed in this species (*2*), which is a possible reason for the conservation of the species’ genome found in previous studies (*24*,*29*).

Although the global prevalence of *C. haemulonii* remains low, nosocomial outbreaks have been reported (*9*,*24*). Nosocomial transmission of *C. haemulonii* was first reported in Kuwait in 2006 (*9*). Because bloodstream infection caused by *C. haemulonii* was rare at the time of the report, the outbreaks were determined by successive isolations of *C. haemulonii* with identical phenotypic characteristics made in the same ward (a neonatal intensive care unit) within a short period of time (3 months), but the outbreaks were not verified by molecular typing. Such molecular methods as ITS sequence typing, random amplified polymorphic DNA analysis, amplified fragment length polymorphism analysis, and, more recently, matrix-assisted laser desorption/ionization time-of-flight mass spectrometry have been applied in *C. haemulonii* studies (*2*,*30*); however, all of those methods have limitations in discriminatory power. Considering the low genetic diversity of *C. haemulonii*, traditional molecular typing assays are not suitable for providing solid evidence for the dissemination of *C. haemulonii*.

WGS-based analysis provides a high-resolution alternative for confirming bacterial and fungal outbreak transmission (*16*,*31*,*32*). Even for species with low genetic diversity, such as *Saprochaete clavata*, this approach can clearly distinguish between sporadic cases and epidemic outbreaks (SNPs <400) (*33*). In this study, we proposed a pairwise SNP difference of ≤50 bp as a criterion for determining clonal cluster cases in *C. haemulonii*, and identified 4 regional clusters that met the criterion. In the absence of medical record evidence, we could not determine whether the case clusters were outbreaks. A previous study by Chow et al. used a 12-bp SNP difference as a cutoff value for interpreting *C. auris* outbreaks (*25*). We suggest that an equally strict pairwise difference might be needed to characterize *C. haemulonii* outbreak events; however, this hypothesis requires further investigation.

*C. auris* has a potent ability to colonize humans and persist in the hospital environment, and biofilm formation is considered the main contributor (*34*). *C. haemulonii* can form dense biofilms (*35*,*36*), which are thought to enhance its capacity to cause regional dissemination and nosocomial transmission. The epidemic cluster events of *C. haemulonii* identified in China had further implications. Several studies have reported that *C. haemulonii* has a low susceptibility to triazoles and amphotericin B (*9*,*37*,*38*). Our study further revealed that antifungal resistance was more obvious among *C. haemulonii* strains from China than among those from other geographic regions (*24*). Moreover, the 4 regional clusters we identified were all caused by antifungal-resistant clones: cluster A was caused by a clone resistant to amphotericin B, cluster B by a clone resistant to 5-fluorocytosine, cluster C by a clone cross-resistant to fluconazole and 5-fluorocytosine, and cluster D by a clone resistant to 3 classes of antifungals. Cluster A was recovered from 2 hospitals located in the same city, suggesting interfacility transmission. Gade et al. reported that 2 strains of *C. haemulonii* isolated from different healthcare facilities in Valencia, Venezuela, were closely related (with only 32 SNPs) (*24*). As with the closely related species *C. auris*, which presents a serious global health threat (*5*,*25*), the emergence of *C. haemulonii* clones with high rates of both transmission and antifungal resistance should be taken as a warning.

A potential limitation of this study is that only a limited number of publicly available genomes were available for *C. haemulonii*, and they were derived from systematic epidemiology surveillances. These isolates, therefore, may not represent real-world *C. haemulonii* distributions globally. To this end, further genomic-based studies need to be conducted with more isolates from different geographic regions.

In conclusion, we studied a total of 94 *C. haemulonii* genomes, including 36 international strains (38.2%) from the National Center for Biotechnology Information Sequence Read Archive database and 58 strains (61.7%) from 23 hospitals in China. We observed 4 phylogenetic clades, 3 of which were identified exclusively in China and exhibited higher antifungal resistance. All fluconazole-resistant strains carried the Y132F substitution in Erg11p. WGS confirmed that the 4 regional cluster cases were caused by specific clones. We additionally noted a potential interfacility transmission within the same city and the spread of multidrug-resistant clones. As with its close relative *C. auris*, *C. haemulonii* should be recognized as a potential threat to global health, and further monitoring and stewardship steps to limit excessive antifungal usage are warranted.

Appendix 1 Additional details on *Candida haemulonii* strains from study of clonal dissemination of antifungal-resistant *C. haemulonii*, China.

Appendix 2 Materials and methods used for study of clonal dissemination of antifungal-resistant *Candida haemulonii*, China.

## References

[1] Lavarde VDF, Saez H, Arnold M, Faguer B. Peritonite mycosique a *Torulopsis haemulonii.* Bull Soc Fr Mycol Med. 1984;13:173–6.

[2] Cendejas-Bueno E, Kolecka A, Alastruey-Izquierdo A, Theelen B, Groenewald M, Kostrzewa M, et al. Reclassification of the Candida haemulonii complex as *Candida haemulonii* (*C. haemulonii* group I), *C. duobushaemulonii* sp. nov. (*C. haemulonii* group II), and *C. haemulonii var. vulnera* var. nov.: three multiresistant human pathogenic yeasts. J Clin Microbiol. 2012;50:3641–51. 10.1128/JCM.02248-12 22952266 PMC3486233

[3] Satoh K, Makimura K, Hasumi Y, Nishiyama Y, Uchida K, Yamaguchi H. *Candida auris* sp. nov., a novel ascomycetous yeast isolated from the external ear canal of an inpatient in a Japanese hospital. Microbiol Immunol. 2009;53:41–4. 10.1111/j.1348-0421.2008.00083.x 19161556

[4] Lockhart SR, Etienne KA, Vallabhaneni S, Farooqi J, Chowdhary A, Govender NP, et al. Simultaneous emergence of multidrug-resistant *Candida auris* on 3 continents confirmed by whole-genome sequencing and epidemiological analyses. Clin Infect Dis. 2017;64:134–40. 10.1093/cid/ciw691 27988485 PMC5215215

[5] Eyre DW, Sheppard AE, Madder H, Moir I, Moroney R, Quan TP, et al. A *Candida auris* outbreak and its control in an intensive care setting. N Engl J Med. 2018;379:1322–31. 10.1056/NEJMoa1714373 30281988

[6] Ben-Ami R, Berman J, Novikov A, Bash E, Shachor-Meyouhas Y, Zakin S, et al. Multidrug-Resistant *Candida haemulonii* and *C. auris*, Tel Aviv, Israel. Emerg Infect Dis. 2017;23:195–203. 10.3201/eid2302.161486 28098529 PMC5324804

[7] Hou X, Xiao M, Chen SC, Wang H, Cheng JW, Chen XX, et al. Identification and antifungal susceptibility profiles of *Candida haemulonii* species complex clinical isolates from a multicenter study in China. J Clin Microbiol. 2016;54:2676–80. 10.1128/JCM.01492-16 27535688 PMC5078542

[8] Lima SL, Francisco EC, de Almeida Júnior JN, Santos DWCL, Carlesse F, Queiroz-Telles F, et al. Increasing prevalence of multidrug-resistant *Candida haemulonii* species complex among all yeast cultures collected by a reference laboratory over the past 11 years. J Fungi (Basel). 2020;6:110. 10.3390/jof6030110 32679832 PMC7558365

[9] Khan ZU, Al-Sweih NA, Ahmad S, Al-Kazemi N, Khan S, Joseph L, et al. Outbreak of fungemia among neonates caused by *Candida haemulonii* resistant to amphotericin B, itraconazole, and fluconazole. J Clin Microbiol. 2007;45:2025–7. 10.1128/JCM.00222-07 17428940 PMC1933024

[10] Kolipinski MC; van UDEN. Torulopsis haemulonii nov. spec., a yeast from the Atlantic Ocean. Antonie van Leeuwenhoek. 1962;28:78–80. 10.1007/BF02538724 14039875

[11] Antony SP, Singh IS, Sudheer NS, Vrinda S, Priyaja P, Philip R. Molecular characterization of a crustin-like antimicrobial peptide in the giant tiger shrimp, *Penaeus monodon*, and its expression profile in response to various immunostimulants and challenge with WSSV. Immunobiology. 2011;216:184–94. 10.1016/j.imbio.2010.05.030 20580462

[12] Pagani DM, Heidrich D, Paulino GV, de Oliveira Alves K, Dalbem PT, de Oliveira CF, et al. Susceptibility to antifungal agents and enzymatic activity of *Candida haemulonii* and *Cutaneotrichosporon dermatis* isolated from soft corals on the Brazilian reefs. Arch Microbiol. 2016;198:963–71. 10.1007/s00203-016-1254-0 27282152

[13] Buck JD. Occurrence of human-associated yeasts in the feces and pool waters of captive bottlenosed dolphins (*Tursiops truncatus*). J Wildl Dis. 1980;16:141–9. 10.7589/0090-3558-16.1.141 6990021

[14] Ferreira N, Belloch C, Querol A, Manzanares P, Vallez S, Santos A. Yeast microflora isolated from brazilian cassava roots: taxonomical classification based on molecular identification. Curr Microbiol. 2010;60:287–93. 10.1007/s00284-009-9539-z 19924478

[15] Glushakova AM, Zheltikova TM, Chernov II. [Groups and sources of yeasts in house dust]. Mikrobiologiia. 2004;73:111–7. 15074050

[16] Sabat AJ, Budimir A, Nashev D, Sá-Leão R, van Dijl J, Laurent F, et al.; ESCMID Study Group of Epidemiological Markers (ESGEM). Overview of molecular typing methods for outbreak detection and epidemiological surveillance. Euro Surveill. 2013;18:20380. 10.2807/ese.18.04.20380-en 23369389

[17] Muñoz JF, Gade L, Chow NA, Loparev VN, Juieng P, Berkow EL, et al. Genomic insights into multidrug-resistance, mating and virulence in *Candida auris* and related emerging species. Nat Commun. 2018;9:5346. 10.1038/s41467-018-07779-6 30559369 PMC6297351

[18] Theodoropoulos NM, Bolstorff B, Bozorgzadeh A, Brandeburg C, Cumming M, Daly JS, et al. *Candida auris* outbreak involving liver transplant recipients in a surgical intensive care unit. Am J Transplant. 2020;20:3673–9. 10.1111/ajt.16144 32530145

[19] Prestel C, Anderson E, Forsberg K, Lyman M, de Perio MA, Kuhar D, et al. *Candida auris* outbreak in a COVID-19 specialty care unit—Florida, July–August 2020. MMWR Morb Mortal Wkly Rep. 2021;70:56–7. 10.15585/mmwr.mm7002e3 33444298 PMC7808709

[20] Bing J, Hu T, Zheng Q, Muñoz JF, Cuomo CA, Huang G. Experimental evolution identifies adaptive aneuploidy as a mechanism of fluconazole resistance in *Candida auris.* Antimicrob Agents Chemother. 2020;65:e01466–20. 10.1128/AAC.01466-20 33077664 PMC7927865

[21] Rybak JM, Sharma C, Doorley LA, Barker KS, Palmer GE, Rogers PD. Delineation of the direct contribution of *Candida auris ERG11* mutations to clinical triazole resistance. Microbiol Spectr. 2021;9:e0158521. 10.1128/Spectrum.01585-21 34878305 PMC8653815

[22] Rybak JM, Barker KS, Muñoz JF, Parker JE, Ahmad S, Mokaddas E, et al. In vivo emergence of high-level resistance during treatment reveals the first identified mechanism of amphotericin B resistance in *Candida auris.* Clin Microbiol Infect. 2022;28:838–43. 10.1016/j.cmi.2021.11.024 34915074 PMC9467277

[23] Xiao M, Chen SC, Kong F, Xu XL, Yan L, Kong HS, et al. Distribution and antifungal susceptibility of *Candida* species causing candidemia in China: An update from the CHIF-NET Study. J Infect Dis. 2020;221(Suppl 2):S139–47. 10.1093/infdis/jiz573 32176789

[24] Gade L, Muñoz JF, Sheth M, Wagner D, Berkow EL, Forsberg K, et al. Understanding the emergence of multidrug-resistant *Candida*: using whole-genome sequencing to describe the population structure of *Candida haemulonii* species complex. Front Genet. 2020;11:554. 10.3389/fgene.2020.00554 32587603 PMC7298116

[25] Chow NA, Gade L, Tsay SV, Forsberg K, Greenko JA, Southwick KL, et al.; US Candida auris Investigation Team. Multiple introductions and subsequent transmission of multidrug-resistant Candida auris in the USA: a molecular epidemiological survey. Lancet Infect Dis. 2018;18:1377–84. 10.1016/S1473-3099(18)30597-8 30293877 PMC6556114

[26] Chow NA, de Groot T, Badali H, Abastabar M, Chiller TM, Meis JF. Potential fifth clade of *Candida auris*, Iran, 2018. Emerg Infect Dis. 2019;25:1780–1. 10.3201/eid2509.190686 31310230 PMC6711235

[27] Tian S, Bing J, Chu Y, Chen J, Cheng S, Wang Q, et al. Genomic epidemiology of *Candida auris* in a general hospital in Shenyang, China: a three-year surveillance study. Emerg Microbes Infect. 2021;10:1088–96. 10.1080/22221751.2021.1934557 34027824 PMC8183536

[28] Naicker SD, Maphanga TG, Chow NA, Allam M, Kwenda S, Ismail A, et al. Clade distribution of *Candida auris* in South Africa using whole genome sequencing of clinical and environmental isolates. Emerg Microbes Infect. 2021;10:1300–8. 10.1080/22221751.2021.1944323 34176429 PMC8253216

[29] Chow NA, Muñoz JF, Gade L, Berkow EL, Li X, Welsh RM, et al. Tracing the evolutionary history and global expansion of *Candida auris* using population genomic analyses. MBio. 2020;11:e03364–19. 10.1128/mBio.03364-19 32345637 PMC7188998

[30] Lehmann PF, Lin D, Lasker BA. Genotypic identification and characterization of species and strains within the genus *Candida* by using random amplified polymorphic DNA. J Clin Microbiol. 1992;30:3249–54. 10.1128/jcm.30.12.3249-3254.1992 1452710 PMC270642

[31] Etienne KA, Gillece J, Hilsabeck R, Schupp JM, Colman R, Lockhart SR, et al. Whole genome sequence typing to investigate the *Apophysomyces* outbreak following a tornado in Joplin, Missouri, 2011. PLoS One. 2012;7:e49989. 10.1371/journal.pone.0049989 23209631 PMC3507928

[32] Lee SC, Billmyre RB, Li A, Carson S, Sykes SM, Huh EY, et al. Analysis of a food-borne fungal pathogen outbreak: virulence and genome of a *Mucor circinelloides* isolate from yogurt. MBio. 2014;5:e01390–14. 10.1128/mBio.01390-14 25006230 PMC4161253

[33] Vaux S, Criscuolo A, Desnos-Ollivier M, Diancourt L, Tarnaud C, Vandenbogaert M, et al.; Geotrichum Investigation Group. Multicenter outbreak of infections by *Saprochaete clavata*, an unrecognized opportunistic fungal pathogen. MBio. 2014;5:e02309–14. 10.1128/mBio.02309-14 25516620 PMC4271555

[34] Horton MV, Johnson CJ, Kernien JF, Patel TD, Lam BC, Cheong JZA, et al. *Candida auris* forms high-burden biofilms in skin niche conditions and on porcine skin. MSphere. 2020;5:e00910–9. 10.1128/mSphere.00910-19 31969479 PMC6977180

[35] Ramos LS, Mello TP, Branquinha MH, Santos ALS. Biofilm formed by *Candida haemulonii* species complex: structural analysis and extracellular matrix composition. J Fungi (Basel). 2020;6:46. 10.3390/jof6020046 32260180 PMC7345111

[36] Sherry L, Ramage G, Kean R, Borman A, Johnson EM, Richardson MD, et al. Biofilm-forming capability of highly virulent, multidrug-resistant *Candida auris.* Emerg Infect Dis. 2017;23:328–31. 10.3201/eid2302.161320 28098553 PMC5324806

[37] Almeida JN Jr, Motta AL, Rossi F, Abdala E, Pierrotti LC, Kono AS, et al. First report of a clinical isolate of *Candida haemulonii* in Brazil. Clinics (São Paulo). 2012;67:1229–31. 10.6061/clinics/2012(10)18 23070353 PMC3460029

[38] Kim MN, Shin JH, Sung H, Lee K, Kim EC, Ryoo N, et al. *Candida haemulonii* and closely related species at 5 university hospitals in Korea: identification, antifungal susceptibility, and clinical features. Clin Infect Dis. 2009;48:e57–61. 10.1086/597108 19193113

