# Supplementary material for: Clonal Dissemination of Antifungal-Resistant Candida haemulonii, China
Source: Emerg Infect Dis. 2023 Mar;29(3):576–84. doi: 10.3201/eid2903.221082 (PMC9973686; doi:10.3201/eid2903.221082)
Supplement: Appendix 2 — Materials and methods used for study of clonal dissemination of antifungal-resistant Candida haemulonii, China. [file 22-1082-Techapp-s2.pdf]

Article DOI: http://doi.org/10.3201/eid2903.221082

Clonal Dissemination of Antifungal-Resistant *Candida haemulonii*, China

Appendix 2

Materials and Methods

Identification

All strains were identified by Autof MS 1000 (Autobio, Zhengzhou, China) and Vitek MS (bioMérieux, Marcy l'Étoile, France) and by sequencing of the rDNA internal transcribed spacer (ITS) region (ABI 3730XL, Thermo Fisher Scientific, Cleveland, OH, USA). The ITS sequences of *Candida haemulonii* CBS 5149^T, *C. duobushaemulonii* CBS 7798^T, and *C. pseudohaemulonii* CBS 10004^T were used as references.

Antifungal susceptibility testing

The in vitro susceptibility of the strains to nine antifungal drugs (fluconazole, voriconazole, itraconazole, posaconazole, anidulafungin, micafungin, caspofungin, 5-fluorocytosine, and amphotericin B) was determined using the commercial chromogenic susceptibility plate Sensititer YeastOne (Thermo Fisher Scientific). MIC values were interpreted according to the CLSI document M27-S3 (*1*). In addition, MIC ≥4 µg/mL was used for interpreting the "resistance" of amphotericin B (*2*). Multidrug resistance was defined as resistance to at least two classes of antifungal agents.

Whole-genome sequencing (WGS)

Genomic DNA was extracted using the Fungi Genomic DNA Extraction Kit (Solarbio Science & Technology, Beijing, China), according to the manufacturer's recommended

protocols. Library construction and genomic sequencing were performed by Novogene Co. (Beijing, China) according to the manufacturer's protocols. Sequencing libraries were generated using the NEBNext Ultra DNA Library Prep Kit for Illumina (New England Biolabs, Ipswich, MA, USA). Each sample was then sequenced using the Illumina NovaSeq6000 platform with 2 × 150-bp reads and 100 × minimum coverage. Raw genome reads are available at the National Centre for Biotechnology Information (NCBI) under the BioProject accession number PRJNA827237.

Accessing publicly available *C. haemulonii* genomes

We searched the NCBI Sequence Read Archive (SRA) database and acquired all *C. haemulonii* genomes available for download till June 15, 2022. All these genomes were in raw reads data format, with average sequencing depth of >50×. Detailed information for these strains could be found in Appendix 2 Table 4.

SNP analysis

Variant calling was performed for all strains of *C. haemulonii* (*3*). A threshold of 0.01 with (Phred score of 20) was used for trimming the raw Illumina sequencing reads. Quality control was performed for sequencing data using the fastx_toolkit 0.0.13 (http://hannonlab.cshl.edu/fastx_toolkit/download.html). Subsequently, paired-end reads were mapped to the *C. haemulonii* BMU5228 reference genome (GenBank assembly accession number: GCA_011426285.1) using BWA 0.7.17. SNPs were analyzed using SAMtools 0.1.18 (*4–6*). The filtering criteria for SNPs were the same as that reported previously (*7*). Gene prediction and annotation of the reference genome were performed using Augustus 3.3.2 (*8*) and eggNOG 5.0 (*9*), respectively.

Phylogenetic and population structure genetic analyses

A phylogenetic tree of whole-genome SNPs was constructed using the SNPs of all strains studied, based on the maximum likelihood method with 1000 ultrafast bootstrap approximation

replicates on the IQ-TREE web server (*10*). We placed the root of the tree to strain B10441 (CBS5149), which was the most ancient *C. haemulonii* strains identified to date in 1962 (all remaining strains were isolated after 2010). An interactive phylogenetic tree was generated using iTOL v5 (*11*). Fastbaps v1.0.1 (*12*) and YMAP V1.0 (*13*) were used for clade typing and copy number variation analysis. The aligned fasta file was used as input for a principal component analysis (PCA) of genetic covariance using the function fasta2genlight in the R package adegenet 1.3–1 (*14*). Moreover, the mating types (MTs) of the strains were further determined as previously described (*3*). In general, for each strain, the coverage depth of their whole genome was calculated using SAMtools from aligned binary alignment map (BAM) file (*6*). Subsequently, by mapping to the corresponding reference genomes, normalized average read depth at MT locus was obtained, and the MT of the strains was further determined.

Karyotype analysis

Karyotypes of 13 isolates with notable aneuploidy or gene CNVs were visualized using the YMAP V1.0 software. The scaffold copy number is shown in a log2 ratio relative to that of the haploid B11899 reference strain (GenBank assembly accession: GCA_002926055.1) on the y-axis, with 1 copy at the midline clipped to show a maximum of 2 copies. The x-axis indicates the positions of reads on each scaffold mapped to the genome of strain B11899. A vertical red line is used to show the position of the *ERG11* gene (Appendix 1 Figure 2).

References

1. Clinical and Laboratory Standards Institute. Reference Method for Broth Dilution Antifungal Susceptibility Testing of Yeasts: Third Informational Supplement M27–S3. Wayne (PA): The Institute; 2008.

2. Pfaller MA, Espinel-Ingroff A, Canton E, Castanheira M, Cuenca-Estrella M, Diekema DJ, et al. Wild-type MIC distributions and epidemiological cutoff values for amphotericin B, flucytosine, and

itraconazole and *Candida* spp. as determined by CLSI broth microdilution. J Clin Microbiol. 2012;50:2040–6. PubMed https://doi.org/10.1128/JCM.00248-12

3. Gade L, Muñoz JF, Sheth M, Wagner D, Berkow EL, Forsberg K, et al. Understanding the Emergence of Multidrug-Resistant *Candida*: Using Whole-Genome Sequencing to Describe the Population Structure of *Candida haemulonii* Species Complex. Front Genet. 2020;11:554. PubMed https://doi.org/10.3389/fgene.2020.00554

4. Li H, Durbin R. Fast and accurate short read alignment with Burrows-Wheeler transform. Bioinformatics. 2009;25:1754–60. PubMed https://doi.org/10.1093/bioinformatics/btp324

5. Li H. A statistical framework for SNP calling, mutation discovery, association mapping and population genetical parameter estimation from sequencing data. Bioinformatics. 2011;27:2987–93. PubMed https://doi.org/10.1093/bioinformatics/btr509

6. Li H, Handsaker B, Wysoker A, Fennell T, Ruan J, Homer N, et al.; 1000 Genome Project Data Processing Subgroup. The Sequence Alignment/Map format and SAMtools. Bioinformatics. 2009;25:2078–9. PubMed https://doi.org/10.1093/bioinformatics/btp352

7. Huang JJ, Chen XF, Tsui CKM, Pang CJ, Hu ZD, Shi Y, et al. Persistence of an epidemic cluster of *Rhodotorula mucilaginosa* in multiple geographic regions in China and the emergence of a 5-flucytosine resistant clone. Emerg Microbes Infect. 2022;11:1079–89. PubMed https://doi.org/10.1080/22221751.2022.2059402

8. Stanke M, Keller O, Gunduz I, Hayes A, Waack S, Morgenstern B. AUGUSTUS: ab initio prediction of alternative transcripts. Nucleic Acids Res. 2006 Jul 1;34(Web Server issue):W435–9.

9. Huerta-Cepas J, Szklarczyk D, Heller D, Hernández-Plaza A, Forslund SK, Cook H, et al. eggNOG 5.0: a hierarchical, functionally and phylogenetically annotated orthology resource based on 5090 organisms and 2502 viruses. Nucleic Acids Res. 2019;47(D1):D309–14. PubMed https://doi.org/10.1093/nar/gky1085

10. Trifinopoulos J, Nguyen LT, von Haeseler A, Minh BQ. W-IQ-TREE: a fast online phylogenetic tool for maximum likelihood analysis. Nucleic Acids Res. 2016;44(W1):W232-5. PubMed https://doi.org/10.1093/nar/gkw256

11. Letunic I, Bork P. Interactive Tree Of Life (iTOL) v5: an online tool for phylogenetic tree display and annotation. Nucleic Acids Res. 2021;49(W1):W293–6. PubMed https://doi.org/10.1093/nar/gkab301

12. Tonkin-Hill G, Lees JA, Bentley SD, Frost SDW, Corander J. Fast hierarchical Bayesian analysis of population structure. Nucleic Acids Res. 2019;47:5539–49. PubMed https://doi.org/10.1093/nar/gkz361

13. Abbey DA, Funt J, Lurie-Weinberger MN, Thompson DA, Regev A, Myers CL, et al. YMAP: a pipeline for visualization of copy number variation and loss of heterozygosity in eukaryotic pathogens. Genome Med. 2014;6:100. PubMed https://doi.org/10.1186/s13073-014-0100-8

14. Jombart T, Ahmed I. adegenet 1.3-1: new tools for the analysis of genome-wide SNP data. Bioinformatics. 2011;27:3070–1. PubMed https://doi.org/10.1093/bioinformatics/btr521

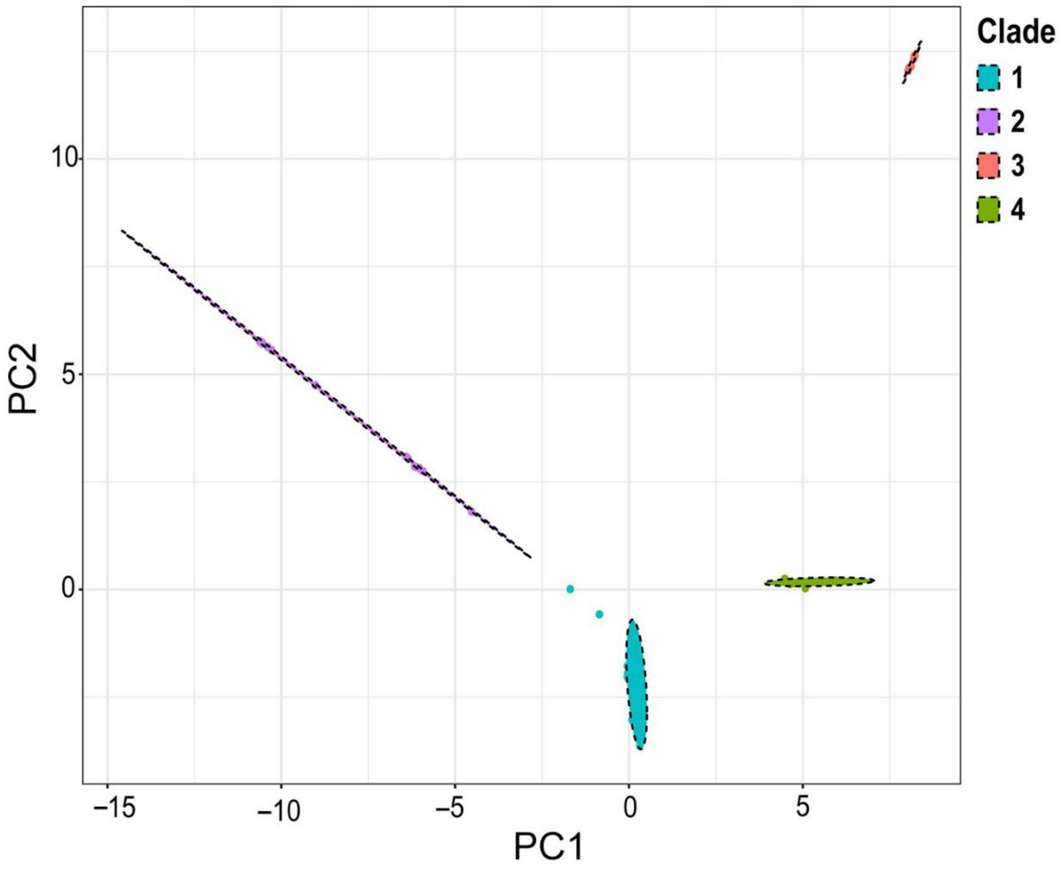

Appendix 2 Figure 1. Population structure differentiation of 4 *Candida haemulonii* clades demonstrated by principal component analysis (PCA).

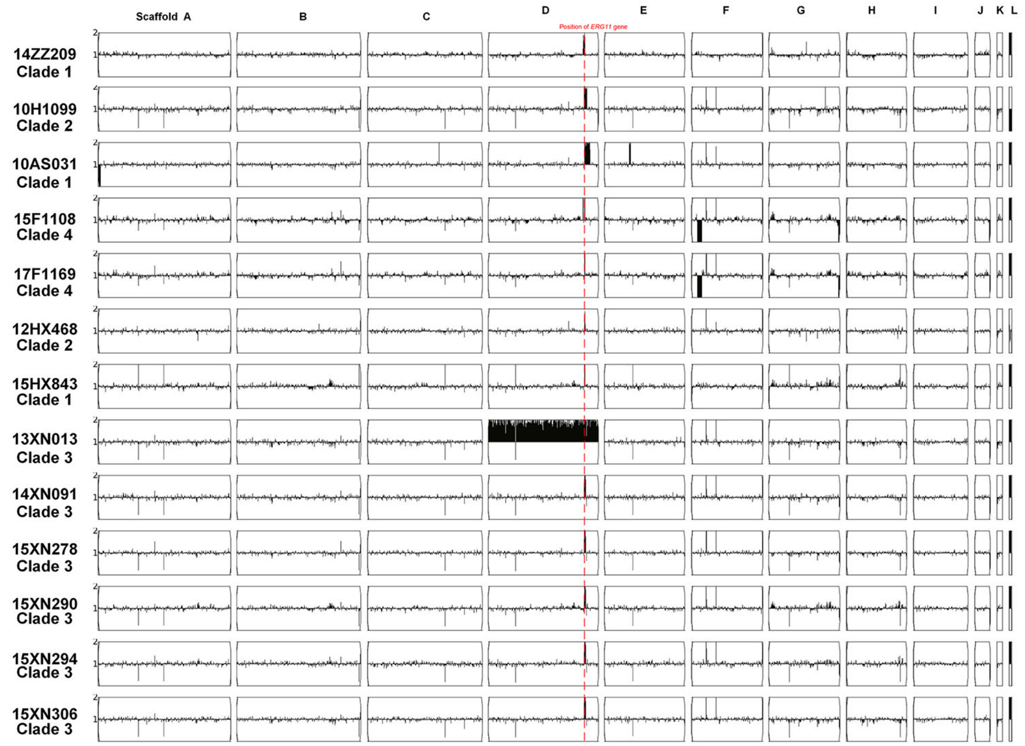

Appendix 2 Figure 2. Copy number variations (CNVs) identified in 13 *Candida haemulonii* isolates.
